# Supplementary material for: The Systems Biology Research Tool: evolvable open-source software
Source: BMC Syst Biol. 2008 Jun 29;2:55. doi: 10.1186/1752-0509-2-55 (PMC2446383; doi:10.1186/1752-0509-2-55)
Supplement: Additional file 1 — SBRT Archive. An archive of the current version of the Systems Biology Research Tool. [file 1752-0509-2-55-S1.zip › sbrt-1.4.0/doc/developers_guide/api/sbrt/kernel/fba/proc/SvInitialPointProcess.html]

SvInitialPointProcess


|  |  |  |
| --- | --- | --- |
| **Overview** \| **Package** \| **Class** \| **Use** \| **Tree** \| **Deprecated** \| **Index** \| **Help** | |  |
| **PREV CLASS**   NEXT CLASS | **FRAMES**    **NO FRAMES**     **All Classes** |  |
| SUMMARY: NESTED \| FIELD \| CONSTR \| METHOD | DETAIL: FIELD \| CONSTR \| METHOD |  |


---


## sbrt.kernel.fba.proc Class SvInitialPointProcess

```
java.lang.Object
  sbrt.kernel.fba.proc.FbaProcess<R>
      sbrt.kernel.fba.proc.CvFbaOptProcess<InitialPointComputer.Results>
          sbrt.kernel.fba.proc.SvInitialPointProcess
```

**All Implemented Interfaces:**: KernelProcess<InitialPointComputer.Results>, MonitoredProcess<InitialPointComputer.Results>

---

``` public final class SvInitialPointProcess extends CvFbaOptProcess<InitialPointComputer.Results> implements MonitoredProcess<InitialPointComputer.Results> ```

This class is used to perform constraint variation initial point experiments.
Initial points are required for random flux vector experiments.

**Author:**
:   This class was written and documented by Jeremiah Wright while in the
    Wagner lab.

**See Also:**: `CdHarFluxVectorProcess`

---

**Constructor Summary**

`SvInitialPointProcess(InitialPointComputer computer)`
  
          Constructs a new constraint variation initial point experiment.


| **Method Summary** | |
| --- | --- |
| `Fluxome` | `getFluxome()`             Returns the fluxome used in this initial point experiment. |
| `ProgressMonitor` | `getProgress()`             Returns a progress monitor for this experiment. |
| `void` | `run(ResultsManager<InitialPointComputer.Results> resultsManager)`             Runs this process and adds the generated results to the provided results manager via its `ResultsManager.addData(Object)` method. |
| `void` | `setConstraintSource(java.lang.Iterable<FluxConstraints> constraintSource)`             Sets the constraint variation input source. |

**Methods inherited from class sbrt.kernel.fba.proc.CvFbaOptProcess**

`restoreConstraints, setConstraints`

**Methods inherited from class sbrt.kernel.fba.proc.FbaProcess**

`toString`

**Methods inherited from class java.lang.Object**

`clone, equals, finalize, getClass, hashCode, notify, notifyAll, wait, wait, wait`

**Constructor Detail**

### SvInitialPointProcess

```
public SvInitialPointProcess(InitialPointComputer computer)
```

:   Constructs a new constraint variation initial point experiment.

    **Parameters:**: `computer` - the computer with which initial points will be computed.


**Method Detail**

### getFluxome

```
public Fluxome getFluxome()
```

:   Returns the fluxome used in this initial point experiment.

    :   **Specified by:**: `getFluxome` in class `FbaProcess<InitialPointComputer.Results>`
    :   **Returns:**: the fluxome used in this initial point experiment.

---


### setConstraintSource

```
public void setConstraintSource(java.lang.Iterable<FluxConstraints> constraintSource)
```

:   Sets the constraint variation input source.

    :   **Parameters:**: `constraintSource` - a source of constraint sets whose effect upon application is to be computed.

---


### getProgress

```
public ProgressMonitor getProgress()
```

:   Returns a progress monitor for this experiment.

    :   **Specified by:**: `getProgress` in interface `MonitoredProcess<InitialPointComputer.Results>`
    :   **Returns:**: a progress monitor for this experiment.

---


### run

```
public void run(ResultsManager<InitialPointComputer.Results> resultsManager)
```

:   Runs this process and adds the generated results to the provided results
    manager via its `ResultsManager.addData(Object)` method.

    First, a set of flux constraints is retrieved from the constraint source
    and applied. Then the initial point is computed and added to the provided
    results manager. Finally, the flux constraints are restored to their
    original values. This process is repeated until the end of the constraint
    source is reached.

    :   **Specified by:**: `run` in interface `KernelProcess<InitialPointComputer.Results>`
    :   **Parameters:**: `resultsManager` - the results manager to which the results will be added.


---


|  |  |  |
| --- | --- | --- |
| **Overview** \| **Package** \| **Class** \| **Use** \| **Tree** \| **Deprecated** \| **Index** \| **Help** | |  |
| **PREV CLASS**   NEXT CLASS | **FRAMES**    **NO FRAMES**     **All Classes** |  |
| SUMMARY: NESTED \| FIELD \| CONSTR \| METHOD | DETAIL: FIELD \| CONSTR \| METHOD |  |


---
